# Secure phasing of private genomes in a trusted execution environment with TX-Phase

**DOI:** 10.1101/gr.280558.125

**Published:** 2025-12

**Authors:** Natnatee Dokmai, Kaiyuan Zhu, S. Cenk Sahinalp, Hyunghoon Cho

**Affiliations:** 1 Department of Biomedical Informatics and Data Science, Yale School of Medicine, New Haven, Connecticut 06510, USA;; 2 Department of Computer Science and Engineering, University of California San Diego, La Jolla, California 92093, USA;; 3 Cancer Data Science Laboratory, National Cancer Institute, National Institutes of Health, Bethesda, Maryland 20892, USA;; 4 Department of Computer Science, Yale University, New Haven, Connecticut 06511, USA

## Abstract

Genotype imputation servers enable researchers with limited resources to extract valuable insights from their data with enhanced accuracy and ease. However, the utility of these services is limited for those with sensitive study cohorts or those in restrictive regulatory environments owing to data privacy concerns. Although privacy-preserving analysis tools have been developed to broaden access to these servers, none of the existing methods support haplotype phasing, a critical component of the imputation workflow. The complexity of phasing algorithms poses a significant challenge in maintaining practical performance under privacy constraints. Here, we introduce TX-Phase, a secure haplotype phasing method based on the framework of trusted execution environments (TEEs). TX-Phase allows users’ private genomic data to be phased while ensuring data confidentiality and integrity of the computation. We introduce novel data-oblivious algorithmic techniques based on compressed reference panels and dynamic fixed-point arithmetic that comprehensively mitigate side-channel leakages in TEEs to provide robust protection of users’ genomic data throughout the analysis. Our experiments on a range of data sets from the UK Biobank and Haplotype Reference Consortium demonstrate the state-of-the-art phasing accuracy and practical runtimes of TX-Phase. Our work enables secure phasing of private genomes, opening access to large reference genomic data sets for a broader scientific community.

Genotype imputation servers (Das et al. 2016) are invaluable tools for the genomics community, providing streamlined analysis pipelines that enhance the quality and resolution of genotype data sets. By utilizing reference panels of high-quality genomes, these servers perform *phasing* to estimate the haplotypes in the user's data, followed by *imputation* to infer missing genotypes not captured in the original genotyping experiment. These servers are especially beneficial for users who lack access to extensive reference panels, sufficient computing resources, or expertise in the necessary data processing steps, enabling them to outsource these tasks to a service provider (SP). However, despite their broad utility, the need to upload private genomic data to a remote server restricts access to these servers for users with particularly sensitive data (e.g., linked to rare health conditions or underrepresented populations) or those working in environments with stringent data sharing constraints stemming from privacy concerns.

Privacy-enhancing technologies (PETs) (Cho et al. 2024) offer a promising approach to expanding access to servers offering genome analysis as a service. This is achieved by enabling the secure processing of users’ data, without requiring complete trust in the SP and their security precautions. For example, recent work (Dokmai et al. 2021) has shown that genotype imputation can be securely and accurately performed within trusted execution environments (TEEs) (Sabt et al. 2015). TEEs are isolated computing environments equipped with hardware/microprocessor-level safeguards that maintain the confidentiality of user data and the integrity of computation, even from other entities with high-privileged access (e.g., administrative access) to the same device. Although cryptographic tools based on homomorphic encryption provide a valuable alternative with strong security guarantees (Kim et al. 2021; Gürsoy et al. 2022), they are less computationally flexible than TEEs, often resulting in notable accuracy loss owing to the algorithmic simplifications needed for practical runtimes (Dokmai et al. 2021).

Unfortunately, none of the existing solutions based on PETs offer a viable secure alternative to existing imputation services, as they fail to support the critical task of phasing. Haplotype phasing (Loh et al. 2016; Browning et al. 2018; Delaneau et al. 2019; Browning et al. 2021; Hofmeister et al. 2023) refers to the task of partitioning an individual's diploid genotypes into a pair of haplotype sequences, one inherited from each parent. Because imputation algorithms depend on patterns of sequence sharing among individuals at the haplotype level through shared lineage (also known as identity by descent [IBD]), the input data must first be phased to ensure accurate imputation. However, phasing, like imputation, is challenging for individual researchers to perform with limited data or computing resources, leading to the widespread use of *imputation servers*, such as the TOPMed and Michigan imputation servers (Das et al. 2016; Taliun et al. 2021), to outsource both tasks. Moreover, phasing is becoming increasingly important as a standalone task to explore various haplotype-level properties of the human genome (e.g., variant combinations, *cis*-regulation, and local ancestry) (Tewhey et al. 2011; Snyder et al. 2015).

Developing a secure method for phasing poses significant challenges. The combinatorial nature of phasing, involving the assignment of alleles to haplotypes across many loci, inherently results in high computational costs, even without privacy considerations. Moreover, in the context of TEEs, the necessity to redesign algorithms to mitigate side-channel leakages (Lou et al. 2021), that is, ensuring that memory access and timing patterns do not expose private information (which we refer to as *oblivious* computation), often introduces substantial computational overhead. This mitigation is essential because a side-channel leakage could be exploited to extract sensitive genomic data processed within a TEE (Brasser et al. 2017; Dokmai et al. 2021). These limitations have, thus far, hindered the practical application of privacy-preserving phasing technologies.

In this work, we introduce TX-Phase, a secure phasing method that enables reference-based phasing of private genomes in remote computing environments. Specifically, we design a solution that leverages TEEs to provide strong security guarantees, incorporating comprehensive mitigations against side-channel leakage while achieving practical accuracy and runtime performance. To this end, we develop novel algorithmic techniques, including compressed reference panels and dynamic scaling of fixed-precision numbers, to make secure computation on haplotype sequences both efficient and as accurate as state-of-the-art phasing tools such as SHAPEIT4 (Delaneau et al. 2019). Building on prior work on TEE-based imputation (Dokmai et al. 2021), the goal of our work is to complete the design of a secure, end-to-end pipeline for genotype imputation servers, thereby expanding the accessibility of such services to the broader research community.

## Results

### Overview of TX-phase

The primary application setting of TX-Phase involves two entities: a user with unphased (diploid) genotype samples and a SP with a large haplotype reference panel. The user seeks to run a reference-based phasing algorithm on their samples using the SP's reference panel. However, the reference panel is assumed to be inaccessible to the user, because of either its large size or strict access controls, preventing local phasing and requiring the SP to perform the analysis on the user's behalf. To support this service, the SP can either use their own computing servers or a provisional third-party cloud infrastructure (e.g., Microsoft Azure). The key challenge addressed by TX-Phase is enabling secure outsourcing of computation while ensuring the user's private data remains protected from unauthorized entities—including the SP, the server operator (whether the SP or a third party), and potential adversaries with unauthorized access. TX-Phase is designed to support this workflow securely, efficiently, and accurately at state-of-the-art levels.

The security of TX-Phase relies primarily on SP's use of a TEE (Sabt et al. 2015). A TEE ensures that the user's data are decrypted and processed only within an isolated computing environment, known as a *secure enclave*, which is protected from the rest of the system and other users (including the SP) through secure hardware and cryptographic protocols. The analysis output is returned to the user via an encrypted channel, ensuring that the SP cannot access the results, even if they coordinate the transfer of encrypted files for usability. Beyond data confidentiality, some TEEs also provide mechanisms for users to verify the integrity of the secure enclave processing their workload. This is achieved through remote attestation (Banks et al. 2021), which allows the user to confirm that the enclave is correctly instantiated and untampered. Although we demonstrate TX-Phase using Intel Software Guard Extensions (SGX) (Costan and Devadas 2016), the most widely adopted TEE framework, our method is compatible with other TEE technologies, such as Intel TDX (Intel Corporation 2022) and AMD SEV (Kaplan et al. 2021).

TX-Phase provides robust security beyond typical TEE applications by offering additional protection against side-channel leakages. A *side channel* (Lou et al. 2021) is an unintended pathway through which information about a user's private data, held within a secure enclave, can be inferred. In particular, timing and memory side channels leak information through variations in operation runtimes or memory access patterns that depend on private data (Fei et al. 2021; Schaik et al. 2024). This is an inherent limitation of SGX and other TEE architectures, often acknowledged but typically left unaddressed in favor of computational performance. Prior research has shown that side channels in genomic analysis algorithms can be exploited to infer private genotypes, even when executed within a TEE (Brasser et al. 2017; Dokmai et al. 2021). TX-Phase eliminates all known timing and memory side channels by introducing algorithmic techniques that ensure constant-time execution and data-independent memory access. This results in what we term an *oblivious phasing* algorithm, which achieves strong security guarantees while maintaining practical performance.

In the following, we highlight two key algorithmic techniques introduced in TX-Phase for efficient oblivious phasing: *compressed haplotype selection* and *dynamic fixed-point arithmetic*. First, compressed haplotype selection leverages a compressed reference panel, based on the M3VCF representation (Das et al. 2016), to accelerate various components of the phasing algorithm by operating only on unique haplotype sequences within each genomic block. This technique significantly reduces the computational costs of (1) querying the positional Burrows–Wheeler transform (PBWT) (Durbin 2014) to identify the reference haplotypes most related to the target sample and (2) filtering the reference panel to construct a conditioned panel for phase estimation.

Next, dynamic fixed-point arithmetic eliminates the need for floating-point operations, which introduce timing side channels (Andrysco et al. 2015). Fixed-point numbers represent fractional numbers using integers by applying a fixed scaling factor and rounding, thereby preventing side-channel leakage through constant-time integer operations. However, traditional fixed-point arithmetic suffers from limited precision. To address this, we introduce dynamically adjustable scaling factors shared among groups of numbers in matrices and vectors. These scaling factors are updated during the analysis to maintain precision based on the magnitude of data values. Our results demonstrate that this approach enables accurate probabilistic inference and sampling in our phasing algorithm. We detail the TX-Phase algorithm and techniques in the Methods, with additional technical discussions provided in Supplemental Notes S1 and S2 in the Supplemental Materials.

**Figure 1.**
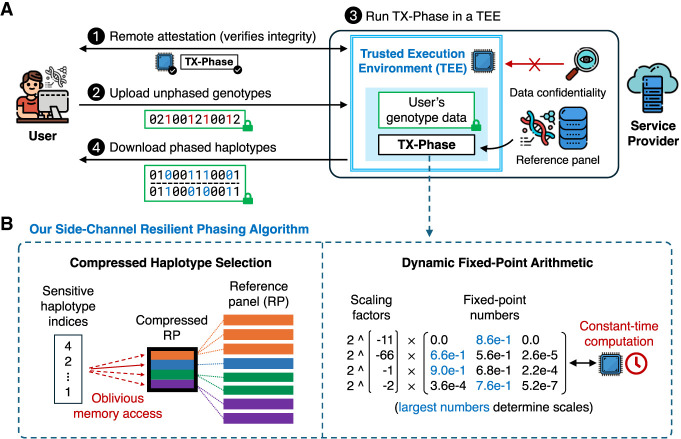
Overview of TX-Phase. We illustrate the workflow of TX-Phase (*A*) and our key algorithmic techniques (*B*). Our workflow proceeds as follows. (*1*) The user and the service provider (SP)’s trusted execution environment (TEE; also called a secure enclave) engage in a remote attestation protocol to verify the integrity of the TEE and the TX-Phase program binary. (*2*) The user establishes a secure channel with the TEE and securely uploads one or more unphased genotype samples via the channel. (*3*) The SP runs TX-Phase inside the secure enclave with a locally held haplotype reference panel to phase the user's genotype samples. (*4*) The user downloads the phased haplotypes from the TEE via the secure channel. TX-Phase offers robust security, ensuring the confidentiality of the user's genomic data from the SP and other users of the system. We introduce two key techniques, *compressed haplotype selection* and *dynamic fixed-point arithmetic*, which enabled us to design a phasing algorithm for TEEs that is efficient, accurate, and safeguarded against the risk of side-channel data leakage. Compressed haplotype selection refers to our novel use of compressed reference panels throughout the algorithm to minimize the overhead of memory-oblivious computation. Dynamic fixed-point arithmetic is our approach for enhancing the precision of fixed-point operations necessary for constant-time computations, achieved by dynamically adjusting the scales of numbers in matrices and vectors. Our results show that both techniques are essential for the secure and practical deployment of TEE-based haplotype phasing.

We summarize the workflow of TX-Phase as follows. First, using a remote attestation protocol, the user verifies the security standing of the TEE platform and confirms the integrity of TX-Phase's program binary within the secure enclave, ensuring it has not been tampered with. Next, the user establishes a secure channel with the enclave to protect sensitive inputs and outputs from leakage or tampering. The user then uploads one or more input genotype samples directly to the enclave via this secure channel. Subsequently, the SP securely phases the input genotypes with TX-Phase inside the enclave while preventing timing and memory side-channel leaks. Optionally, the SP can also perform genotype imputation on the phased genotypes within the enclave using a previously developed algorithm (e.g., SMac) (Dokmai et al. 2021). Finally, the user retrieves the phased (and optionally imputed) haplotypes from the enclave via the secure channel. We illustrate our workflow and key techniques in Figure 1.

### TX-Phase provides state-of-the-art phasing accuracy

We first evaluate the phasing accuracy of TX-Phase on 424 parent-offspring trios from the UK Biobank (UKB) and compare with two state-of-the-art phasing tools: SHAPEIT4 (Delaneau et al. 2019) and Eagle2 (Loh et al. 2016). TX-Phase is closely modeled after SHAPEIT4's algorithm, which we comprehensively redesigned to achieve the side-channel resilience property for secure deployment in a TEE (see Methods) (Supplemental Note S1). Eagle2 represents the current method of choice in most existing imputation servers, including the TOPMed and Michigan imputation servers. We focused our analysis on Chromosome 20 (19,959 genetic variants) and sampled reference panels of varying sizes (20,000, 40,000, 100,000, 200,000, and 400,000 haplotypes) from the remaining UKB cohort. Phasing accuracy is measured by the *switch error rate* (SER), which refers to the proportion of consecutive heterozygous sites that are incorrectly phased compared to Mendelian phasing. Additional details on the data set and evaluation setting are provided in Methods.

**Figure 2.**
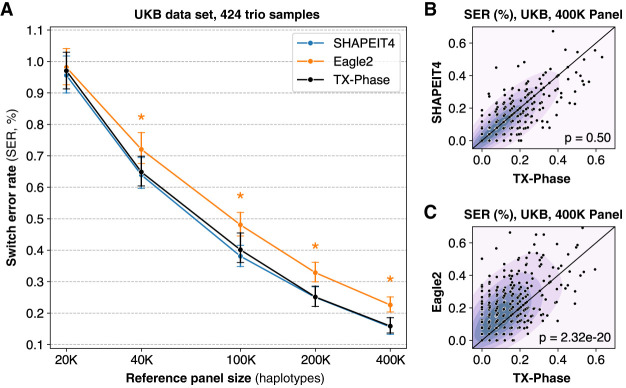
TX-Phase obtains state-of-the-art phasing accuracy on the UK Biobank (UKB) data set. (*A*) We compared the accuracy of TX-Phase with two standard phasing methods, SHAPEIT4 (Delaneau et al. 2019) and Eagle2 (Loh et al. 2016), on 424 trio samples from the UKB (genotyping array data; Chromosome 20) using subsets of the remaining cohort of varying sizes as the reference panel. Accuracy is measured by switch error rates (SERs) with trio-based phasing as the ground truth. Markers indicate the mean SERs, and error bars represent 95% confidence intervals. Asterisks denote highly significant differences (two-sided Wilcoxon signed-rank test, *P* < 1 × 10^−10^) between TX-Phase and Eagle2. The same statistical tests were performed between TX-Phase and SHAPEIT4, which did not result in any significant difference. We also compare the per-sample SERs of TX-Phase against those of SHAPEIT4 (*B*) and Eagle2 (*C*) for the 400,000 reference panel. The background heatmap visualizes marker density. For clarity, the display region is limited to [0, 0.7] for both axes. We show the *P*-values from the two-sided Wilcoxon signed-rank test, which suggest that TX-Phase significantly outperforms Eagle2 while obtaining comparable accuracy with SHAPEIT4. Consistent results are observed when phasing a sample from the Genome-in-a-Bottle Consortium using the 1000 Genomes and Haplotype Reference Consortium reference panels (Supplemental Figure S1).

We observed nearly identical mean accuracies between TX-Phase and SHAPEIT4 across all reference panel sizes without any statistically significant difference (two-sided Wilcoxon signed-rank test [WSR] *P* > 0.21) (Fig. 2A). Because of the stochastic nature of the phasing algorithm, small differences in accuracy between the two methods are observed for individual samples (Fig. 2B), but this difference was not skewed toward either method (e.g., WSR *P* = 0.50 for the 400,000 panel). Furthermore, TX-Phase significantly outperformed Eagle2 in accuracy for all but the smallest 20,000 reference panel (WSR *P* < 1.7 × 10^−11^ in all cases, with *P* = 2.32 × 10^−20^ for the 400,000 panel) (Fig. 2A,C). This suggests that TX-Phase could improve both the security and accuracy of the current phasing workflow on existing imputation servers.

For additional validation, we evaluated TX-Phase on the Ashkenazi trio from the Genome-in-a-Bottle (GIAB) Consortium data set using the 1000 Genomes Phase 3 (1KG) data set (5008 haplotypes from 2504 subjects) and the Haplotype Reference Consortium (HRC) data set (54,330 haplotypes from 27,165 subjects) as reference panels. Consistent with the UKB results, TX-Phase achieved accuracy comparable to that of SHAPEIT4 and outperformed Eagle2 for both the 1KG and HRC reference panels (Supplemental Fig. S1). These results demonstrate that TX-Phase provides state-of-the-art phasing accuracy despite its reliance on fixed-point arithmetic owing to the security constraints.

### TX-Phase is practical in running time and memory usage

**Figure 3.**
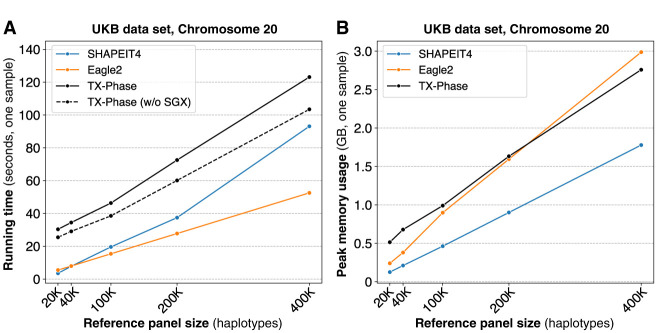
TX-Phase maintains practical running times for phasing individual samples while providing enhanced security. We report the per-sample running times (*A*) and peak memory usages (*B*) of TX-Phase, SHAPEIT4, and Eagle2 for phasing the UKB genotyping array data set (Chromosome 20; 19,959 variants) with varying reference panel sizes up to 400,000 haplotypes. Additionally, we plot the running times of TX-Phase without SGX for comparison, in which our program is executed in a conventional (unprotected) computing environment. The results demonstrate that TX-Phase effectively minimizes the overhead of side-channel resilient computation in TEEs, achieving practical performance comparable to existing tools, or is only marginally more costly (e.g., by less than a factor of three) on large reference panels.

We next evaluate the computational costs of TX-Phase with respect to both running time and peak memory usage and compare them with SHAPEIT4 and Eagle2. Because of the additional computational costs introduced by executing a program in a TEE and our side-channel protection techniques, some slowdown is expected for TX-Phase compared with the existing non-TEE methods. Nevertheless, we observed practical running times for TX-Phase on the UKB data set across all reference panel sizes (Fig. 3A). For example, TX-Phase took 2 min (123 sec) to phase Chromosome 20 using the 400,000 reference panel, reflecting an increase of 1.3-fold and 2.3-fold in running time compared with SHAPEIT4 (93 sec) and Eagle2 (53 sec), respectively. Extrapolating these results, we estimate that phasing a whole-genome sample (e.g., with 800,000 variants) with the 400,000 haplotype reference panel would take 82 min using TX-Phase compared with 63 min using SHAPEIT4 and 35 min using Eagle2.

To quantify the overhead of TEEs, we also timed the execution of TX-Phase outside the secure enclave for comparison. We observed small additional runtime costs for TX-Phase, for example, 19% on the largest reference panel (Fig. 3A). In comparison, running SHAPEIT4 or Eagle2 within the enclave through the Gramine framework (Tsai et al. 2017), a framework for running existing software in a TEE without modifications, resulted in larger overhead costs of 71% and 43% on the largest panel, respectively (Supplemental Fig. S2). Notably, TX-Phase was even faster than SHAPEIT4 on the largest panel when both were executed in a TEE, illustrating the effective design of TX-Phase that minimizes the TEE overhead. We further note that directly deploying existing phasing tools in a TEE introduces side-channel vulnerabilities and thus is not recommended; TX-Phase represents a practical alternative with robust protection against such risks.

The peak memory usage of TX-Phase was comparable to Eagle2 and 1.6 times that of SHAPEIT4 for the largest reference panel (Fig. 3B). TX-Phase's memory usage remained <3 GB in all settings, which allows it to be deployed on low-cost servers with standard memory resources. Note that phasing the whole genome does not increase the memory usage of TX-Phase because fixed-size genomic windows are processed independently, which leads to memory usage that depends primarily on the size of the window and the number of haplotypes in the reference panel.

### Compressed haplotype selection enables scalable performance

TX-Phase introduces novel algorithmic techniques based on compressed reference panels to minimize the cost of oblivious computation in phasing. To demonstrate the significance of these techniques, we compared TX-Phase with its modified version that uses the original (uncompressed) reference panel following the same approach as the original SHAPEIT4 algorithm but with side-channel protection.

We focus on two analysis steps in the selection of conditional haplotypes (i.e., reference haplotypes that are closest to the phasing estimates of the target sample) that pose a computational bottleneck given a large reference panel: *PBWT querying* and *haplotype filtering*. PBWT querying refers to the task of retrieving the indices of reference haplotypes with the longest suffix matches with the target sequence at different sequence positions using the PBWT data structure. Haplotype filtering refers to the subsequent step of constructing a reduced reference panel consisting only of the chosen haplotypes for use in the hidden Markov model (HMM)–based inference algorithm for phase estimation (Methods). TX-Phase accelerates both these steps using a compressed reference panel, which allows us to operate over the space of unique haplotypes within each genomic block and avoids full scans over the original panel, which is overwhelmingly costly when performed obliviously to hide sensitive access patterns.

**Figure 4.**
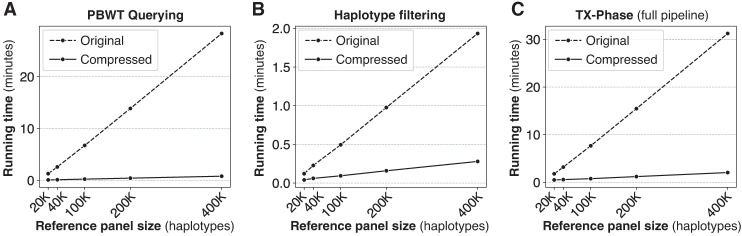
Our compressed haplotype selection techniques are essential for TX-Phase's computational efficiency. We compare TX-Phase (compressed) with an alternative side-channel resilient implementation without compression (original), which more closely follows SHAPEIT4's approach, with respect to their running times. We report the per-sample running time for phasing Chromosome 20 using the UKB data set across varying reference panel sizes. The results are summarized for different components of the algorithm, including PBWT querying (*A*) and haplotype filtering (*B*), as well as the full phasing pipeline (*C*). Our compressed algorithms substantially reduce the running time of TX-Phase, which otherwise becomes prohibitive for large reference panels.

Our results show that our compression techniques are pivotal to TX-Phase's practical running times. Based on the largest panel of 400,000 haplotypes, TX-Phase is more than 35 times faster than the alternative approach without our compression techniques for PBWT querying (Fig. 4A) and seven times faster for haplotype filtering (Fig. 4B), which together lead to a 15 times faster running time for the entire phasing pipeline (Fig. 4C). For whole-genome phasing (with 800,000 variants) using the 400,000 reference panel, we estimate that TX-Phase would result in a running time of 1.4 h compared to 21 h without our compression techniques.

### Dynamic fixed-point arithmetic ensures accurate phasing

We designed TX-Phase to use only constant-time, fixed-point arithmetic operations to prevent the timing side channels introduced by floating-point operations. This is achieved with high precision and efficiency by dynamically updated scaling factors for groups of fixed-point numbers (e.g., rows or columns of a matrix; see Methods). Previous work on TEE-based imputation (Dokmai et al. 2021) proposed performing probability calculations in the logarithmic domain to minimize precision loss at the cost of extra computation required for addition and subtraction of probabilities, which become nonlinear operations in the log space. Although this approach was shown to be effective for the imputation task, we observed that the same approach would lead to prohibitive running times for phasing owing to the larger dimensions of the matrices involved, which introduce more additions relative to other operations. Our complexity analysis suggests that adopting the log-domain approach would result in a 100-fold slowdown of TX-Phase performance (Supplemental Tables S1, S2).

**Figure 5.**
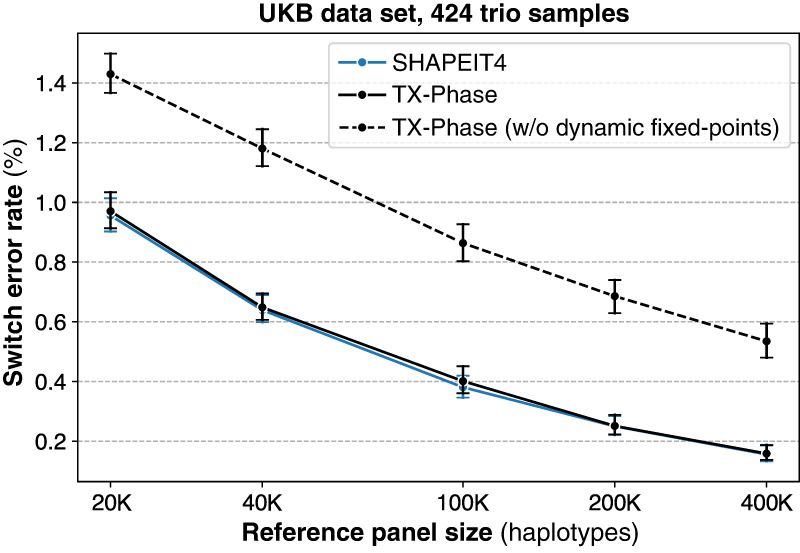
Our dynamic fixed-point arithmetic techniques ensure accurate phasing of TX-Phase under precision constraints. We compare the phasing accuracy of TX-Phase, measured by the SER, with an alternative implementation (with standard fixed points) without our dynamic scaling techniques for fixed-point matrices and vectors. The evaluation is conducted on 424 trio samples (Chromosome 20) from the UKB data set, using reference panels of varying sizes from the remaining cohort. Markers indicate the mean SERs, and error bars represent 95% confidence intervals. We also include the accuracies of SHAPEIT4 for comparison, which are shown to be comparable with those of TX-Phase. Without our dynamic fixed-point techniques, the accuracy of TX-Phase significantly deteriorates.

Comparing TX-Phase with an alternative implementation that omits our dynamic scaling technique, instead using an optimized number of fractional bits for fixed points with the most accurate results (52 bits for 64-bit integers in our setting), revealed that our approach substantially reduces the SER on the UKB data set consistently across different reference panel sizes (Fig. 5). For instance, with the 400,000 reference panel, TX-Phase achieves an average SER of 0.16%, compared to 0.55% without our technique, representing an improvement of more than threefold. The accuracy loss without dynamic scaling typically results from integer underflows, in which entire distributions are incorrectly flattened to a uniform distribution owing to small probabilities turning into zeros, thus introducing problems in the sampling process of the phasing algorithm. Our technique allows TX-Phase to closely match the accuracy of SHAPEIT4, despite TX-Phase's reliance on fixed-point numbers with limited precision.

## Discussion

Our method TX-Phase demonstrates the practicality of secure haplotype phasing powered by TEE technologies, with state-of-the-art phasing accuracy and robust side-channel protection. Together with an existing tool for imputation (Dokmai et al. 2021), our work represents the completion of an end-to-end TEE-based solution for genotype imputation servers, encompassing both phasing and imputation workflows.

Although our experiments focused on single-sample phasing for comparison, processing an input data set with multiple samples can be easily supported and accelerated using multiple CPUs, for example, by creating multiple instances of the TX-Phase secure enclave to process samples in parallel. We confirmed that the overhead associated with such parallelization is minimal for TX-Phase (Supplemental Fig. S3). SHAPEIT4 incorporates additional optimizations to process multiple samples more efficiently; similarly extending our approach to minimize redundant computation across samples, for example, by sharing the compressed PBWT data structure, is a meaningful direction for future work. Furthermore, our method can be extended to target rare variants in the output haplotypes following the approach introduced by SHAPEIT5 (Hofmeister et al. 2023).

Another future direction is to explore broader applications of the algorithmic techniques introduced in TX-Phase. Our compressed PBWT data structure and associated operations have the potential to accelerate sequence search in large genomic databases beyond the TEE setting, particularly when integrated with PBWT-based methods for tasks such as phasing (Loh et al. 2016; Delaneau et al. 2019; Browning et al. 2021), imputation (Browning et al. 2018), and IBD detection (Zhou et al. 2020). Moreover, our techniques for oblivious computation could serve as building blocks for the development of additional TEE-based tools, enabling confidential analysis of sensitive biomedical data.

## Methods

### Review of Intel SGX TEE

Intel SGX (Costan and Devadas 2016) is one of the most mature TEE platforms for user-level applications. Intel SGX employs hardware root-of-trust, cryptographic techniques and decentralized trust to ensure the confidentiality and integrity of data and code within a secure enclave. Confidentiality ensures that no unauthorized processes can access protected information inside the enclave during runtime. Integrity guarantees that data and codebases verified by Intel and the remote user cannot be modified by unauthorized processes during runtime. This allows sensitive applications to be deployed in untrusted computing environments, even when the operating system might be compromised. Remote users can verify the security status of SGX hardware and the authenticity of runtime applications via a process called remote attestation (Banks et al. 2021) and can deny participation before uploading sensitive input if the security status is not up to standard, for example, if there are unmitigated vulnerabilities.

Despite promising initial applications in genomic analysis (Kockan et al. 2020; Dokmai et al. 2021), the practical adoption of this technology will require broader algorithmic development, together with careful consideration of its security guarantees and the incorporation of complementary safeguards. The research community has discovered a variety of security vulnerabilities in its architecture over the years (Fei et al. 2021; Schaik et al. 2024). Although most security vulnerabilities have been patched in the latest Intel CPUs, side-channel leakage, in which private information is exposed through externally observable metrics such as timing or memory access patterns, remains inherent to the design of current TEEs, leaving method developers responsible for mitigations. Our key approach in TX-Phase is to thoroughly eliminate the possibility of side-channel leakage at the *algorithmic* level.

### TX-Phase's security model

TX-Phase's setting involves two primary participants: the SP and the user. SP deploys TX-Phase within a secure enclave in a TEE-enabled computing environment. The user verifies the enclave and submits unphased genotypes as input samples to the SP. The SP then uses TX-Phase, alongside a large reference panel of haplotype sequences (which is accessible only to the SP), to phase the genotype samples and returns the phased haplotypes to the user. This workflow is illustrated in Figure 1A.

We consider a setting in which SP has full control over the computing environment, including privileged access to the operating system and physical devices. This includes typical setups in which the SP manages their own infrastructure or in which the service is hosted on a virtual machine within a commercial cloud platform. A potential attacker, with the same level of access as the SP, may attempt to extract the user's genotypes by tampering with or inspecting various components of the computing environment. Leveraging TEEs, TX-Phase ensures robust protection of the user's input data against this attacker. In certain TEE platforms with integrity protection, such as Intel SGX and Intel TDX, TX-Phase also safeguards the integrity of the phasing results. We assume that the TEE's security status is up to date, allowing TX-Phase to mitigate side-channel vulnerabilities beyond those addressed by the TEE's standard security model. Importantly, we assume the SP acts in good faith and is committed to preventing such attack by implementing additional security measures that may further strengthen the system such as access control, scheduled data deletion, and regular software updates. TX-Phase should be seen as a complementary tool that significantly enhances the security offered by these practices, enabling collaboration between the SP and the user to achieve optimal security outcomes.

### Side-channel vulnerabilities of TEEs

The following are two main categories of side-channel vulnerabilities in TEEs demonstrated in the literature.

#### Timing side channels

Computational operations can take different amounts of time depending on the values of the input. For instance, owing to the architectural design of CPUs, floating-point multiplications involving subnormal (near-zero) numbers can take significantly longer than those involving large numbers (Andrysco et al. 2015). This timing discrepancy can be exploited by side-channel attacks, such as port contention (Aldaya et al. 2019), to infer information about the private input. Prior work has demonstrated that this can result in the leakage of sensitive genotypes during imputation workflows (Dokmai et al. 2021). For example, a key step in the probabilistic inference over a HMM of haplotype sequences (Delaneau et al. 2019) involves updating a probability distribution after observing a genotype at a specific position. The timing of this update can reveal whether a user's genotype matches the reference genotype.

#### Memory-access side channels

Querying a data structure using private information, such as accessing an array with private indices, or performing logical branching based on a private condition can both result in secret-dependent memory access patterns. An external process observing the memory access patterns of an SGX application, using techniques like Prime+Probe (Brasser et al. 2017), can exploit these differences to infer private inputs. In haplotype phasing, a key step involves selecting a subset of reference panel haplotypes closest to the user's genotypes. This subset could potentially be leaked through memory side channels and thus reveal information about the user's genotypes.

Many software countermeasures to both timing and memory side channels in TEE have been proposed in the literature (Fei et al. 2021), each with various trade-offs between security, performance, and ease of use. TX-Phase not only builds upon these general strategies but also introduces new algorithmic techniques to obtain a scalable solution for TEE-based phasing with comprehensive side-channel mitigation.

### Overview of our side-channel mitigation strategies

#### Memory-oblivious algorithms

TX-Phase employs memory-oblivious computation techniques to ensure that local processes (including the host operating system) monitoring the memory access patterns of the program cannot infer any sensitive information about the input data. For array lookups, TX-Phase uses a *linear-scanning* oblivious RAM (ORAM) (Chang et al. 2016), which hides access patterns by uniformly accessing all items in an array. Despite its suboptimal asymptotic complexity compared with more sophisticated methods (Stefanov et al. 2018; Tople et al. 2019), its simplicity and lack of runtime/storage overhead from internal data structures make this approach the most efficient option for the moderately sized arrays and read-focused tasks common in TX-Phase. Oblivious sorting in TX-Phase is implemented using Bitonic sort (Batcher 1968). Oblivious filtering (i.e., selection of a subset of elements in an array) is achieved by reordering the selected elements to the front of the array (via oblivious sorting) and then truncating the array. In addition to these low-level routines, to make oblivious algorithms scalable for large data sets (e.g., the reference panel in phasing), TX-Phase introduces compressed data structures and associated operations for haplotypes (detailed in the section “Our compressed haplotype selection for accelerating oblivious phasing”).

#### Deterministic control flow

To ensure that the control flow of TX-Phase (such as if or for statements) is not affected by the sensitive input, we make the control flow fully deterministic and input-independent. Specifically, an if statement is replaced with a deterministic multiplexer that executes all possible code paths and selects the outcome based on the sensitive condition. Similarly, for loops are structured to always iterate up to a fixed upper bound. However, such redundancy can quickly become intractable. Our solution is to minimize additional computational costs by restructuring algorithms for more streamlined control flow and maximal partitioning of computations involving sensitive and nonsensitive data.

#### Constant-time instructions

Certain CPU instructions in phasing algorithms are known to be input dependent in running time, particularly floating-point arithmetic and integer division (Andrysco et al. 2015). To mitigate this timing discrepancy, we follow the method of Andrysco et al. (2015) in using only constant-time CPU instructions for TX-Phase's implementation of code involving sensitive data. This includes using fixed-point primitives (based on integers, whose running time is input independent) to represent real numbers and implementing constant-time integer long division using bitwise operations. In contrast to a previous work on genotype imputation (Dokmai et al. 2021), which proposed performing fixed-point arithmetic in log space for precision, TX-Phase develops a new framework based on dynamically scaled fixed-point numbers, which allows TX-Phase to obtain computational efficiency and state-of-the-art phasing accuracy (see section “Our dynamic fixed-point arithmetic for accurate constant-time computation”).

#### Secure typing of sensitive data

Following previous work on imputation (Dokmai et al. 2021), we adopt a secure typing system in Rust programming language to ensure that all sensitive input data are appropriately protected in TX-Phase. This is achieved by creating a wrapper around standard types and overriding their input-dependent subroutines. The secure types enforce a set of rules in which (1) any subroutines and computation involving them must be carefully implemented to be constant-time and data-oblivious in memory access; (2) computation of secure types must always result in a secure type; and (3) exposure of secure types into nonsecure types must be explicit. The benefit of this approach is that any violation of the security rules can be detected at the time of compilation, thus speeding up software development and making the resulting code more reliable. In our work, we extend the secure types from the previous work to include secret indices that can only be used to access arrays via ORAM or other data-oblivious algorithms.

#### Comparison to existing side-channel mitigation techniques

TX-Phase mitigates side channels through algorithmic and code transformations optimized for the workflows and data structures used in haplotype phasing. In contrast, generic side-channel mitigation techniques in the literature (Fei et al. 2021) are agnostic to algorithm design and data flow. Although the generic approaches are easier apply to existing software, such techniques often address only partial attack vectors, for example, by detecting anomalous attacker behavior (Gruss et al. 2017; Shih et al. 2017), or impose high runtime overheads (Ahmad et al. 2019; Brasser et al. 2019). For instance, OBFUSCURO (Ahmad et al. 2019), a generic tool providing a comparable level of protection to TX-Phase, incurs an average 83-fold runtime overhead in its experimental applications and up to 231-fold overhead for matrix multiplications, a key operation in phasing algorithms. Our experimental results additionally demonstrate that applying existing side-channel mitigation techniques lead to substantial computational overhead or accuracy loss in phasing, both of which are addressed by TX-Phase.

### TX-Phase's reference-based phasing algorithm

TX-Phase adopts the reference-based phasing approach of a state-of-the-art tool SHAPEIT4 (Delaneau et al. 2019), which we thoroughly redesigned to remove all known timing and memory access side channels while maintaining efficient and accurate performance (see Supplemental Note S1). This approach utilizes the Markov chain Monte Carlo (MCMC) algorithm to progressively improve the estimated phased haplotypes of the target sample through iterative sampling. We briefly outline the computation involved in each sampling iteration below.

In each iteration, a small but targeted *conditioned reference panel* is constructed, which is a subset of haplotypes from the original reference panel that share the longest identity-by-state (IBS) segments with the current estimate of the phased haplotypes. This haplotype subset is chosen efficiently using the PBWT data structure (Durbin 2014). Given the conditioned panel, we use a variant of the Li–Stephens HMM (Li and Stephens 2003), which models each haplotype sequence as a mosaic of the reference haplotypes, to compute the transition probabilities over the phased genotypes between adjacent genomic segments, representing the current belief over the target phases. These probabilities are then used to sample a new pair of phased haplotype estimates, provided as the input to the next iteration. The state space of the HMM is augmented to include both the reference panel and the segmented haplotype model of the target sample. The latter represents all possible phased haplotypes as paths through a *genotype graph*, which includes short nonoverlapping haplotype segments as nodes. Each segment covers a small number of heterozygous sites (three by default) to allow the enumeration of all possible phases of each segment for inference.

The MCMC algorithm for phasing proceeds through several different stages of the sampling process: (1) *initialization*, in which a heuristic strategy is used to guess the initial phases of the target sample to use as an initial search point; (2) *burn-in*, in which some number of sampling iterations is performed to move the initial estimate closer to a desired sample from the posterior distribution over the phases; (3) *pruning*, in which adjacent phased haplotype segments with high confidence are merged to reduce the search space; and (4) *main*, in which the final, maximum-likelihood phases are determined via the Viterbi posterior decoding algorithm. TX-Phase inherits the sampling procedure from SHAPEIT4, involving multiple rounds of alternating burn-in and pruning stages before performing main iterations to obtain the final output.

### Our compressed haplotype selection for accelerating oblivious phasing

The scale of reference panels poses a significant challenge to the efficiency of phasing applications, particularly in side-channel-resilient algorithms, in which computational redundancy is critical for obscuring runtime patterns linked to sensitive data. TX-Phase addresses this challenge by leveraging the positional PBWT (Durbin 2014) on compressed reference panels for haplotype selection, substantially reducing the search space during the oblivious construction of the conditioned reference panel for HMM inferences.

TX-Phase's compression approach is inspired by fast imputation methods using a compressed reference panel, specifically the M3VCF encoding (Das et al. 2016), to significantly reduce the operational size of large reference panels, for example, by two orders of magnitude for the HRC or UKB data sets. In this encoding scheme, the original reference panel is divided into short contiguous genomic “blocks,” with the haplotypes within each block compressed by retaining only the *unique* haplotypes observed. Next, TX-Phase applies PBWT to compressed reference panel blocks, enabling an efficient search for the *S* “nearest neighbors” of the current estimate of the phased haplotypes based on the longest common suffix (LCS) at each randomly selected genomic position across the phasing window. The aggregate of these nearest neighbors across all positions forms the conditioned reference panel. To ensure that the nearest neighbors maintain the longest LCS across independent compressed block regions, TX-Phase introduces a method for updating the global LCS at only the infrequent transitional points between block regions, thus improving the efficiency while preserving the accuracy of haplotype selection.

Crucially, by combining a compressed data structure and our side-channel mitigation strategies (see section “Overview of our side-channel mitigation strategies”), TX-Phase is able to obliviously construct the conditioned reference panel while ensuring that the runtime and memory access patterns remain independent of sensitive user genotypes. The key steps of the algorithm are outlined below. Further details, including pseudocode for the compressed PBWT operations, are provided in Supplemental Note S2, with visual illustrations in Figure 1B and Supplemental Figure S4 and notations defined in Supplemental Tables S4 and S5.

#### Step 1: PBWT on compressed blocks and estimated haplotype insertion

Our improved PBWT algorithm independently transforms each compressed reference panel block. Subsequently, the estimated haplotypes of the target samples are retroactively and data-obliviously inserted into the PBWT structures for neighbor searches. By separating nonsensitive data (the reference panel) from sensitive data (the estimated haplotypes), this approach allows PBWT structures to be reused across different MCMC iterations and target samples while preventing side-channel leakage. Unlike the standard PBWT procedure in SHAPEIT4, our method encodes the transformation in a compact prefix tree representation, with one prefix tree per compressed block. This compact representation groups haplotypes by common prefixes, enabling efficient memory storage and searches for reference haplotypes that share the longest LCS with the estimated haplotypes.

In this prefix tree structure, each node represents a group of haplotypes sharing common prefixes from the start of the block, whereas each leaf corresponds to a unique haplotype within the block. Nodes at each position are lexicographically sorted by their “reverse prefix” (i.e., the suffix at reverse genomic positions), referred to as the *positional prefix order* in PBWT. *Divergences*, which capture the LCS between adjacent haplotypes in the positional prefix order, are maintained between neighboring nodes. The estimated haplotypes are partitioned into block regions and inserted into the prefix trees in the correct positional prefix order, with divergences updated separately from the tree. The prefix tree representation and insertion process are visualized in Supplemental Figure S4 (panels 1 and 2).

#### Step 2: nearest neighbor candidate searches

Leveraging the compressed PBWT structures, our algorithm rapidly identifies nearest-neighbor candidates by locating haplotypes with the longest block-level LCS (i.e., within the block region) at the search position. This candidate selection step eliminates the need to compute the global LCS (i.e., across the entire phasing window) for *all* haplotypes to find the true *S* nearest neighbors later in step 3, which is a costly process.

The search proceeds as follows. At each (randomly selected) search position, haplotypes are searched in groups, as captured by the tree nodes. Haplotypes within the same node share a common prefix and therefore have the same block-level LCS by definition, allowing them to be collectively identified as candidates. To find nodes with the longest block-level LCS, the algorithm performs local searches by leveraging PBWT's locality, in which nodes with longer block-level LCS are clustered together, and the LCS can be computed on the fly from the divergences encoded in the tree. To ensure that all true *S* nearest neighbors are included in the candidate set, the algorithm guarantees that at least *S* candidates are identified in total. The candidate searches are visualized in Supplemental Figure S4 (panel 3).

#### Step 3: finding the *S* nearest neighbors

The *S* nearest neighbors are ranked among the candidates based on their global LCS relative to the estimated haplotypes. To achieve this efficiently, our method computes the global LCS for all reference haplotypes *once* at the starting position of the block region and then uses this information to rank haplotype candidates across *any* search positions within the block. This process relies on two key observations. First, haplotype candidates can be grouped by the nodes where they reside, with these nodes ordered according to their block-level LCS. Because a shorter block-level LCS implies a shorter global LCS, the haplotypes with the shortest global LCS will come from the last node. As a result, only haplotypes from this last node need to be ranked by their global LCS and eliminated until exactly *S* nearest neighbors remain. Second, to rank haplotypes within the same node, we only need to compute their global LCS at the starting position of the block, as haplotypes within the same node share a common prefix. The global LCS array at the starting position is recursively computed by expanding the block-level LCS from the preceding prefix trees and combining these values to form the global LCS. Importantly, this method conceals the path of the estimated haplotypes within the prefix trees. First, haplotypes within each node are ranked by their global LCS for *all* nodes in the tree, which is efficiently performed using an oblivious merge-sort algorithm in which the merge operations follow the structure of the prefix tree. Then, the appropriate nodes in the path of the estimated haplotypes are obliviously selected to eliminate nonnearest haplotype candidates. This process is visualized in Supplemental Figure S4 (panel 4).

#### Step 4: conditioned reference panel construction

After identifying the *S* nearest neighbors at all search positions, the sets of nearest neighbors are combined to form the global set of conditioned haplotypes using an oblivious set union operation. This is accomplished by representing the membership of haplotypes in the reference panel as a bitmap (a binary vector), and obliviously setting the bits associated with the nearest neighbors (using linear-scanning ORAM) across all positions. The resulting set is then used as a filter to construct a conditioned reference panel from the compressed reference panel by applying an oblivious filtering algorithm.

### Our dynamic fixed-point arithmetic for accurate constant-time computation

Another key challenge in secure TEE-based phasing is to perform probabilistic calculations accurately without using floating-point numbers, which expose timing side channels (Andrysco et al. 2015; Dokmai et al. 2021). Our phasing algorithm relies heavily on probabilistic calculations involving large matrices and vectors, for example, in the HMM inference step, in which the transition probabilities between adjacent haplotype segments are calculated. Previous works have used fixed-point numbers equipped with constant-time integer operations to address this issue (Andrysco et al. 2015; Dokmai et al. 2021). However, fixed points generally have low precision, which could result in catastrophic underflows, in which small probabilities turn into zeros. This in turn reduces the accuracy of phasing, as shown in Figure 5. Although encoding probabilities in log space can offer better precision, this leads to a prohibitive performance overhead in phasing (see Supplemental Tables S1, S2).

To address these limitations, TX-Phase introduces *dynamic fixed-point arithmetic*, which can be viewed as a hybrid of fixed-point and floating-point schemes, combining the constant-time properties of the former with the improved precision of the latter. Our key idea, also illustrated in Figure 1B and Supplemental Figure S5, is to dynamically maintain an additional scaling factor on a row (or column) basis based on the observation that most of the matrices in the phasing algorithm encode a probability distribution in each row (or column). The scaling helps to retain small numbers in the distribution with minimal impact on the desired calculations, as the scaling factors can be easily accounted for when renormalizing the distribution. We determine the scaling factor as the power of two that would make the largest number in the row (or column) between 0.5 and one after scaling. Multiplying or dividing a vector by this factor can be performed efficiently by applying bit-shift operations to the integers representing the fixed points.

Standard arithmetic operations can be performed on our dynamically scaled matrices with additional steps to ensure the scales are consistent. For instance, matrix addition requires that the corresponding rows are shifted to the same scale prior to addition, in which the output scale is chosen to minimize precision loss. Element-wise multiplication of two matrices involves multiplying two matrices element-wise and then adding the exponents of the scaling factors of the corresponding rows. Column-wise summation of a matrix requires that all rows be brought to the highest scale within the matrix to minimize precision loss. After each operation on a matrix, we update the scaling factors to maintain the fixed scale of the largest number in each row.

### Data preprocessing and evaluation settings

To benchmark the accuracy and efficiency of TX-Phase, we utilized the UKB data set (976,754 haplotypes from 488,377 subjects) commonly employed for reference-based phasing benchmarks. We focused our analysis on Chromosome 20, containing 19,959 markers. For cross-validation, we withheld 424 mother–father–child trio samples from the data set to serve as target samples. The ground-truth phases were determined by trio phasing using the “trio-switch-rate” plugin in the bcftools software (https://github.com/samtools/bcftools), which excludes from evaluation all sites that are phase-ambiguous, such as those in which all three individuals are heterozygous. Reference panels were constructed by downsampling the full, phased UKB data set in varying sizes (20,000, 40,000, 100,000, 200,000, and 400,000 haplotypes) to assess the impact of reference panel size on accuracy and efficiency. All types of variants, including single-nucleotide polymorphisms and structural variants (insertions and deletions), were included in the benchmarks.

For additional validation, we used the 1KG data set (5008 haplotypes from 2504 subjects) and the HRC data set (54,330 haplotypes from 27,165 subjects) as reference panels, with the Ashkenazi trio from the GIAB data set as the target sample. We focused our analysis on shared markers between reference panels and the target sample on Chromosome 20: 77,593 markers between 1KG and GIAB and 66,070 between HRC and GIAB. To collect multiple measurements, we ran TX-Phase and SHAPEIT4 with 100 random seeds on the same GIAB target sample. Eagle2, being a deterministic algorithm, consistently produces the same output.

The state-of-the-art phasing methods used for comparison include SHAPEIT4 (Delaneau et al. 2019) and Eagle2 (Loh et al. 2016). SHAPEIT4 serves as a direct comparison as TX-Phase closely follows its phasing algorithm, whereas Eagle2 represents the tool currently used by the Michigan and TOPMed imputation servers. We used the latest versions of SHAPEIT4 (v5.1.1; now part of SHAPEIT5 releases) and Eagle2 (v2.4.1) with default parameters. TX-Phase shares the default SHAPEIT4 parameters, with the addition of a parameter for minimum heterozygous rates in the target samples (used to calculate the size of the phasing window), set to 10% by default.

Reference panels were compressed to M3VCF format for TX-Phase using Minimac3 (Das et al. 2016) with the default parameters, while remaining in BCF format for SHAPEIT4 and Eagle2. Because TX-Phase addresses phasing and does not impute missing genotypes, these were removed from the target samples. To ensure a fair accuracy comparison with SHAPEIT4, which imputes missing genotypes as a postprocessing step after phasing, imputed genotypes were removed from its output. For Eagle2, we used the ‐‐noImpMissing flag to disable imputation. Missing sites do not provide any information about the underlying haplotypes and do not affect the phasing results for the observed sites. The TEE-based imputation algorithm (Dokmai et al. 2021) can be applied after TX-Phase to support the imputation step if needed.

All experiments were conducted on an Ubuntu server equipped with an Intel Xeon Gold 6426Y processor with SGX version 2, 16 physical CPU cores (hyperthreading disabled for SGX security), and 128 GB of enclave page cache (EPC). Timing and peak memory usage (based on maximum resident set size) were measured using the /usr/bin/time command, which yielded highly consistent measurements across different samples (Supplemental Table S3). Applications running in SGX utilized Gramine (Tsai et al. 2017), a library OS for executing existing binaries within an SGX enclave.

Our experiments did not include measurements for remote attestation and secure data transfer over the network. This is because these steps take negligible amounts of time compared with the phasing algorithm. Remote attestation takes <0.2 sec using the Intel SGX data center attestation primitives (DCAP) protocol (Intel Corporation 2019) and the Intel provisioning certification service (PCS) as the attestation server. Similarly, the cost of secure data transfer of unphased samples (e.g., about 800,000 SNPs in the UKB) and the resulting phased haplotypes between the TEE and user takes only a few milliseconds per sample (based on 400 KB of data per sample and a gigabit ethernet connection with 125 MB/sec bandwidth).

### Implementation details

TX-Phase is implemented in Rust programming language, featuring memory and thread safety and thus preventing the most common security vulnerabilities in software such as memory leaks and race conditions. TX-Phase is deployed in the Gramine framework (Tsai et al. 2017), a library OS for deploying existing program binaries in an SGX enclave without modifications. TX-Phase's default phasing parameters closely mirror those of SHAPEIT4's to produce the same level of phasing accuracy. Both the user's input to TX-Phase and its output are in VCF format, which are securely transmitted between the user and the TX-Phase application using an encryption key known only to the user and the secure enclave. As a result, the data cannot be accessed by any other entity, including the SP, who may coordinate the transfer of the encrypted input/output for usability. The reference panels are preprocessed into the M3VCF format using Minimac3 (Das et al. 2016). Note that the implementation of TX-Phase is agnostic of the TEE frameworks, so it can likewise be deployed in AMD SEV, Intel TDX, and other SGX frameworks. The strong typing system and constant-time implementation are built on the Rust timing-shield library (https://www.chosenplaintext.ca/open-source/rust-timing-shield/).

### Data sets

The UKB genotype array data set can be accessed via the UKB research analysis platform (RAP) at https://ukbiobank.dnanexus.com. RAP is available to all researchers that are collaborating on an approved in-progress UKB project. The 1000 Genomes Project Phase 3 data set is publicly available and can be accessed through the International Genome Sample Resource (IGSR) website at http://ftp.1000genomes.ebi.ac.uk/vol1/ftp/phase3/. The HRC data set is available upon request from the European Genome-phenome Archive (EGA; https://ega-archive.org/) under accession number EGAS00001001710. The GIAB data set is publicly available and can be accessed through the National Institute of Standards and Technology (NIST) website at https://ftp-trace.ncbi.nlm.nih.gov/giab/ftp/data/.

### Software availability

Our open-source implementation of TX-Phase is available as Supplemental Code and at GitHub (https://github.com/hcholab/txphase).

## Supplemental Material

Supplement 1

Supplement 2
